# Hospital use of young children in Switzerland: A nation-wide study based on a complete survey over 4 years

**DOI:** 10.1186/1472-6963-8-267

**Published:** 2008-12-20

**Authors:** Franziska V Schoeni-Affolter, Marcel Widmer, André Busato

**Affiliations:** 1 Institute for Evaluative Research in Orthopedic Surgery, MEM Center, University of Bern, Stauffacherstrasse 78, Bern, Switzerland

## Abstract

**Background:**

Young children are known to be the most frequent hospital users compared to older children and young adults. Therefore, they are an important population from economic and policy perspectives of health care delivery. In Switzerland complete hospitalization discharge records for children [<5 years] of four consecutive years [2002–2005] were evaluated in order to analyze variation in patterns of hospital use.

**Methods:**

Stationary and outpatient hospitalization rates on aggregated ZIP code level were calculated based on census data provided by the Swiss federal statistical office (BfS). Thirty-seven hospital service areas for children [HSAP] were created with the method of "small area analysis", reflecting user-based health markets. Descriptive statistics and general linear models were applied to analyze the data.

**Results:**

The mean stationary hospitalization rate over four years was 66.1 discharges per 1000 children. Hospitalizations for respiratory problem are most dominant in young children (25.9%) and highest hospitalization rates are associated with geographical factors of urban areas and specific language regions. Statistical models yielded significant effect estimates for these factors and a significant association between ambulatory/outpatient and stationary hospitalization rates.

**Conclusion:**

The utilization-based approach, using HSAP as spatial representation of user-based health markets, is a valid instrument and allows assessing the supply and demand of children's health care services. The study provides for the first time estimates for several factors associated with the large variation in the utilization and provision of paediatric health care resources in Switzerland.

## Background

Young children, known to need special health care [1], are an important population from economic and policy perspectives of health care delivery. In the U.S. annual national hospital discharge surveys [2,3] and collections of longitudinal hospital care data (Healthcare Cost and Utilization Project) exist and track development of hospitalization rates for adults and children. Emergency admissions for acute common childhood illnesses[4], particularly respiratory and gastro-intestinal diseases, are known to be the most common reason for children's hospitalization within the first years of age. Other studies reveal in addition to these medical reasons, that variation in hospitalization rates for children can also be attributed to non-medical factors, including area-specific hospital practice patterns, availability of beds, variation in access to ambulatory care [5], socio-demographic factors and, last but not least, environmental quality of life.

The influence of socio-demographic and geographical factors as well as the supply of medical care on the use of children's health care resources has received little attention in Switzerland. The potential importance of studying such factors for the quality as well as for the economics of health care is substantial in the second most expensive health care system of the world with still rising costs [6]. Due to the fact that the Swiss health care is organized on a cantonal basis (Switzerland is divided into 26 federal states), most analyses are done within cantonal boundaries, and few analyses exist, which overview the system of children's hospital care in the country as a whole. One of the problems associated with the estimation of regional availability of health care is the fact that patients often travel beyond their cantonal borders to seek care, especially those living in remote and/or non-urban areas. The formation of meaningful health service areas as unit of analysis is therefore an important issue. It allows control for supply and demand-induced utilization phenomena in each area with its own characteristics. Instead of investigating children's health care on a cantonal basis, we therefore propose for the first time a new spatial model by delineating specific pediatric hospital service areas [HSAP] from the inpatient stationary data with standard methods [7,8], describing the real health markets for these children [9].

The aim of this article is to provide estimates of demand- and supply-related factors that account for variations in hospitalization rates of young children in Switzerland based on small area analysis of medstat which represent aggregated ZIP codes as the smallest units of analysis. The formation of the alternative spatial model of HSAP allows for relevant clustering effects of the small areas within these population-based health markets (e.g. variation in thresholds for stationary admission resulting from availability of beds, outpatient facilities with the possibility of monitoring a child for a short period of time).

## Methods

Stationary hospitalization rates per 1000 children, age 0 to <5 in each medstat [aggregated ZIP code area] were calculated. Ambulatory and short stay consultations provided by outpatient clinics are defined and coded by the hospitals as discharges from a stay lasting less than 24 h and were calculated as ambulatory/short stay consultation rates per 1000 children. Due to the fact that children's hospitalizations have a unique geography [9] Pediatric hospital service areas (HSAP) were created with standard methods according to the Dartmouth atlas of healthcare [7,8,10] and the detailed descriptions by Klauss [10]. The aim was the delineation of user-based health markets based on stationary hospitalization data. The model based analysis then allows respecting the individual strategies for hospitalization typical for each HSAP.

### Data

Complete stationary and ambulatory outpatient hospital discharge data over the years 2002 to 2005 for children < 5 years of age were used, excluding healthy newborns not requiring special care and all discharge records for patients not living in Switzerland (1,321 hospitalizations). Each record represents the discharge data of an individual stationary or outpatient hospitalization classified by gender, age, medstat of residence and medstat of hospitalization respectively. This master file was provided by the Swiss federal statistical office and included the most recent data available. Medstat as smallest unit was used to prevent the possible identification of any patient and, hence, to guarantee the protection of privacy due to the strict confidentiality laws in Switzerland. Additional indications about the reason for hospitalization (diagnosis) and the respective length of stay were available. Data from the 2000 census provided information on demographic attributes of the population in each medstat. Geographical attributes of each medstat concerning urbanity were also available from the Swiss federal statistical office and were matched to the medstat of residence:

- Urbanity:

• Urban and suburban areas:

• Isolated villages

• Rural areas

- Main language spoken in medstat:

• German/Romanic

• French

• Italian

### Data analysis and statistics

The investigation included descriptive statistics of means and/or medians, depending on the distribution of the data, and standard deviations. Non-parametric tests were used to compare means due to the fact that the data were not normally distributed.

The stationary hospitalization rate per 1000 children at medstat level was defined as the main outcome variable and analyzed using a general linear model. Variances in the intercepts and slopes were analyzed and reduced by adding the following explaining variables:

• HSAP

• Classification according to urbanity of patient's medstat

• Classification according to main language [German, French, Italian] of patient's medstat

• Year of hospitalization

• Male/female ratio in the population

• Swiss/non-Swiss ratio in the population

• Amount of ambulatory/short stay care provided by hospital's outpatient clinic as ambulatory/short stay consultations/1000 children in medstat.

Further analyses revealed significant first order interaction terms of the main language with other explanatory variables therefore language-stratified analyses were additionally performed comprising the same set of explanatory variables except language and the related interaction terms.

HSAP, year, language region and urbanity types were treated as classification variables and continuous explanatory variables were centred to facilitate parameter interpretation. Results for categorical data were interpreted as adjusted group means (LSM: least squared means) with 95% confidence intervals (CI95). The Bonferroni procedure was used to adjust for the problem of multiple comparisons. Residual analyses, performed to validate the statistical procedures, showed no evidence of assumption violations for the models used in the analysis. SAS 9.1 (SAS Institute Inc., Cary, NC, USA) was used for all analyses and the level of significance was set at 0.05 throughout the study.

For the graphical output the GIS-compatible vector files for Swiss census regions (MicroGIS Ltd., Baar, Switzerland) were used [11].

## Results

### Hospital discharges

**Figure 1:**
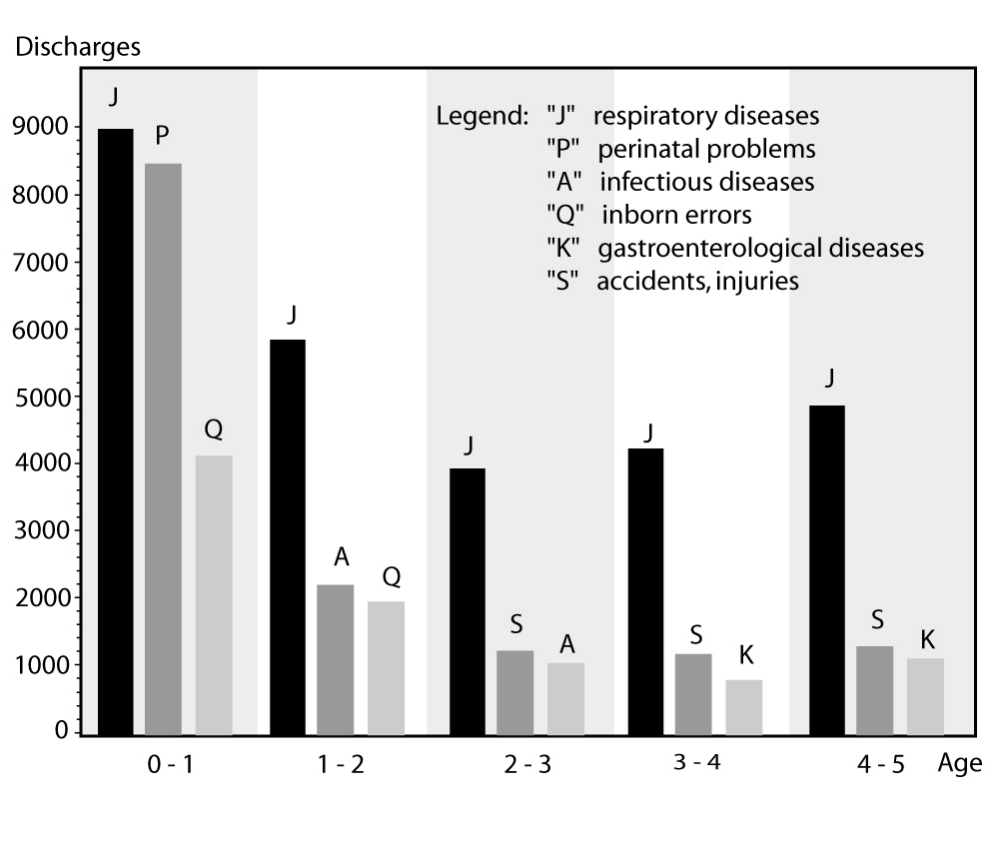
**Most frequent diagnoses in chidern <5 years of age**.

The total number of children's stationary hospital discharges (0 – 4 years of age) for the year 2002 through 2005 was 105,720 (due to lack of coding for ICD10, 1910 records were excluded from the study). Significantly more discharges were found in 2004 and 2005 (2002 = 24,816; 2003 = 25,062; 2004 = 28,178; 2005 = 27,664). 53.8% were emergency admissions requiring treatment within 12 hours (compared to 36% emergency admissions for adults). Over the four years 25.9% (ICD10 "J: respiratory problems" = 27,417) children were diagnosed with respiratory problems (ICD10 "Q: inborn errors" = 8,976 [8.4%]; ICD10 "P: perinatal problems" = 8,675 [8.2%]) [figure 1]. Language stratified analyses of hospitalizations show that in Italian speaking regions 31.5% of the children were diagnosed with respiratory problems, compared to 27.3% in the French and 24.8% in the German speaking regions of Switzerland.

### Stationary Hospitalization rates

The hospitalization rate [hosp-rate] per 1000 children per year varied substantially between the different urbanity types of medstats, language regions and population characteristics [table 1]. Boys were significantly more often admitted to hospital than girls. Significantly more children living in urban or suburban areas were admitted to hospitals than those living in village or rural medstats. Higher hospitalization rates were found for non-Swiss residents compared to Swiss residents. A significant association was observed between hospitalization rate and language region: Children in Italian speaking regions are 1.5 times more likely to be hospitalized than children living in German speaking regions. The overall mean length of stay [LOS] was 4.77 days and significantly longest in the German speaking part [mean = 4.86 days, SD = 12.1] compared to French [mean = 4.68 days, SD = 10.2; p = 0.0243] and Italian speaking parts of the country [mean = 3.9 days, SD = 7.1; p < 0.001].

**Table 1:** Study population and hospitalization discharges per 1000 children on medstat level.

	**Population**	**Hospital discharges per 1000 children per year**		
		**mean [median]**	**range**	**SD**

**Total**	387'181	66.1 [61.8]	16.2 – 252.9	25.1

**Sex**				

female	198'734	57. 8^a ^[54.6]	10.0 – 224.7	24.1
male	188'447	74.0^b ^[68.9]	21.9 – 281.2	29.2

**Rurality**				

Inner city	92'183	74.2^a ^[70.0]	17.5 – 172.3	29.7
Village	162'641	68.3^b ^[64.7]	16.2 – 238.0	24.7
Rural areas	132'357	60.9^b ^[57.5]	21.7 – 252.9	22.4

**Residential status**				

Swiss resident	286'612	61.2^a ^[57.9]	10.7 – 217.9	21.1
Non Swiss resident	100'569	86.6^b ^[77.4]	9.2 – 255.5	43.2

**Language region**				

German	274'467	62.2^a ^[58.3]	16.2 – 160.1	21.4
French	97'679	77.6^b ^[70.6]	32.1 – 252.9	32.9
Italian	15'035	89.9^c ^[89.4]	57.5 –129.1	19.9

^**a, b, c **^Values with different superscripts have significantly different means [p < 0.05]

Because respiratory diseases are the most frequent reason for hospitalizations, we analyzed the data for these indications across urbanity types stratified by language regions. Highest respiratory hospitalization rates were found again in the Italian part in all three urbanity strata (respiratory hosp-rate: urban or suburban areas = 32.9; village areas = 30.3; rural areas = 24.2 per 1000 children) compared to the French (respiratory hosp-rate: urban or suburban areas = 23.1; village areas = 22.3; rural areas = 19.8 per 1000 children) and German parts (respiratory hosp-rate: urban or suburban areas = 18.2; village areas = 15.6; rural areas = 14.1 per 1000 children). The LOS [length of stay], in contrast to the overall LOS, is not significantly different between French (mean = 3.27 days) and Italian regions (mean = 3.02 days; p = 0.124). German regions have significantly longer stays for respiratory problems (mean = 3.83 days; p < 0.0001) compared to the other two areas.

### Ambulatory/outpatient consultations

**Figure 2:**
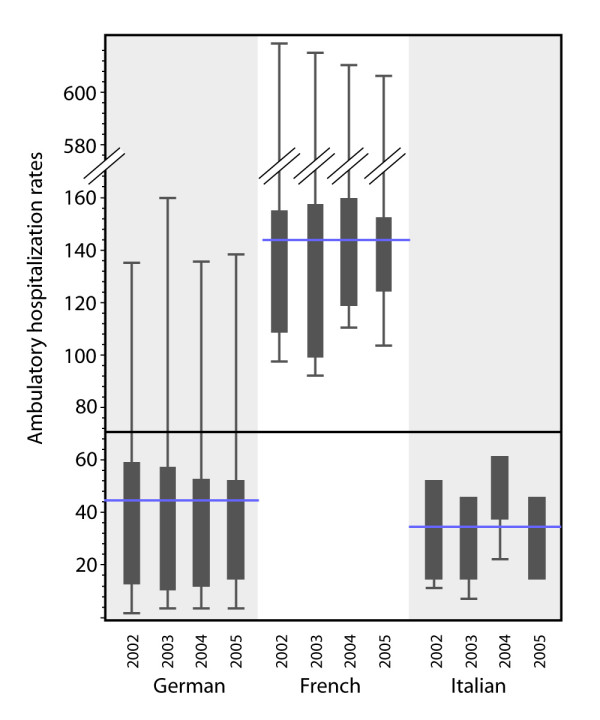
**Ambulatory hospitalization rates in different language regions over 4 years (standard deviation and 95% CL, horizontal lines represent the overall mean in the different language strata)**.

We analyzed the ambulatory services of children's medical care, which is thought to decrease the stationary hospitalization rate. Ambulatory treatment performed by the hospital's outpatient clinics was calculated as discharges for ambulatory or short stay consultations per 1000 children in medstat and year. Language stratified data showed that most ambulatory/outpatient's consultations happened in French speaking regions (mean: 153.8; median 138.2 visits/1000 children). In German and Italian regions the ambulatory consultations amounted to 48.5 visits/1000 children (median: 23.3) and 38.1 visits/1000 children (median: 42.3) respectively [figure 2]. The medstats of one HSAP showed very high ambulatory outpatient consultation rates with a mean of 430.7 visits/1000 children (median: 530.2). Between the different urbanity types no significant difference exists concerning the distribution of ambulatory visits in outpatient clinics.

### HSAP and medstat characteristics

**Figure 3:**
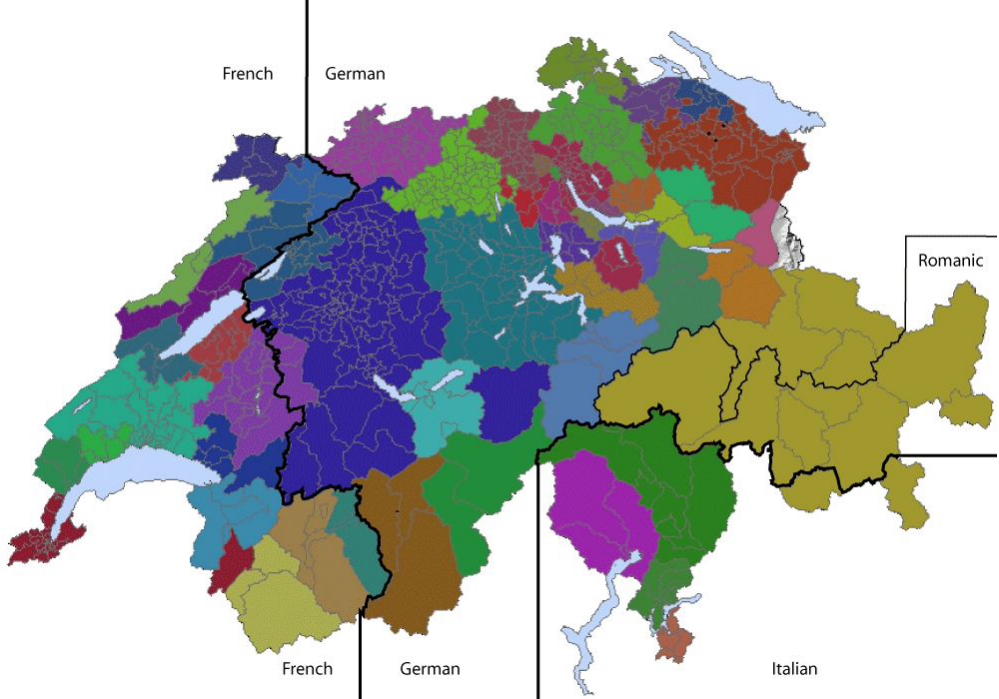
**Delineation of 37 HSAP in Switzerland; small areas within each HSAP represent medstats**.

37 HSAP were delineated from 105,720 hospital discharge data (four years) of 604 medstats [figure 3] each of which included one or more hospitals for treating children. Every HSAP represents therefore a health market for children. 17 HSAP were created in the German [17,263 discharges/year], 16 in the French [7,706 discharges/year] and 4 HSAP in the Italian speaking part [1,461 discharges/year]. The fraction of locally treated patients was 68.3%.

According to the classification for urbanity we found 97 urban or suburban medstats [24% of children's population], 245 medstats with larger village characteristics [42% of children's population] and 262 rural medstats [34% of children's population]. 26.0% of the children's population were non Swiss residents.

### Multiple regression analyses of hospitalization rates

In this study the stationary hospitalization rate on the medstat level was used as outcome variable. We evaluated two general linear models, one with HSAP (R^2 ^= 0.38%) and the other with "canton" (R^2 ^= 0.26%) as clustering unit. The higher R^2 ^of the model with HSAP indicates better model fit with HSAP as clustering unit and therefore it was used in all subsequent models. All variables evaluated in bivariate analyses (see table 1) and shown to be significant were included in the subsequent models. Due to significant interaction terms a stratified analysis by language was performed. In the final model the value of intercept indicates the hospitalization rate at the mean of all continuous explanatory variables for rural region and 2005 as year of reference [classification variables] in the respective stratum. Adjustments for socio-demographic factors (gender ratio and Swiss/non Swiss ratio) and LOS were implemented in the model. Age adjustment was not possible, because the census data included no precise age-related information, only strata information (0 to <5 years of age).

Urbanity shows a significant effect in the German and Italian regions. Urban or suburban areas are associated with significantly higher hospitalization rates than country-side regions [table 2]. Compared to 2005 significantly fewer hospitalizations were registered in 2002 and 2003 for the German part and in 2003 for the French part of Switzerland. In the Italian part no significant difference was found concerning the annual hospitalization rates (not in table). HSAP has a significant effect on the hospitalization rate in the German and French part. In the Italian speaking part only one HSAP was significantly associated with higher hospitalization rates.

**Table 2:** Least square means [LSM] of hospitalization rates across urbanity types stratified by language regions

	**Urbanity**	**Difference in LSM**	**Adjusted CL95**	**Adj. P**
**German**	Inner city vs. Isolated village	9.9	6.9 – 14.8	**<0.0001**
	
	Inner city vs. Country side	17.8	14.9 – 21.6	**<0.0001**
	
	Isolated village vs. Country side	7.9	5.7 – 10.7	**<0.0001**

**French**	Inner city vs. Isolated village	-1.2	-9.2 – 6.8	n.s.
	
	Inner city vs. Country side	0.9	-8.2 – 9.9	n.s.
	
	Isolated village vs. Country side	2.1	-5.3 – 9.4	n.s.

**Italian**	Inner city vs. Isolated village	12.88	0.16 – 25.61	**0.0471**
	
	Inner city vs. Country side	49.86	34.16 – 65.57	**<0.0001**
	
	Isolated village vs. Country side	36.98	25.07 – 48.88	**<0.0001**

In the French part higher ambulatory hospitalization rates are associated with significantly lower stationary hospitalization rates. Yet, in German and Italian regions, where ambulatory outpatient's hospitalizations are not as common, no such association was found. The lower ratio of Swiss residents to non-Swiss residents in a medstat is significantly associated with higher hospitalization rates in the German and French regions [table 3].

**Table 3:** Parameter estimates of continuous explanatory variables on language stratified data

**German regions **(R^2 ^= 0.36)^a^	**Estimate**	**SE**	**P**
Intercept	45.3	6.7	**<0.0001**

Ambulatory hospitalization rate per 1000 children in medstat	0.01	0.009	n.s.

Length of stay	0.02	0.21	n.s.

Ratio of boys to girls	4.4	5.3	n.s.

Ratio Swiss to non Swiss residents	-0.29	0.1	**0.0001**

**French region **(R^2 ^= 0.41)^a^			

Intercept	187.60	23.18	**<0.0001**

Ambulatory hospitalization rate per 1000 children in medstat	-0.08	0.02	**0.0006**

Length of stay	-1.2	0.57	**0.0431**

Ratio of boys to girls	-8.3	18.4	n.s.

Ratio Swiss to non Swiss residents	-1.04	0.44	**0.0290**

**Italian region **(R^2 ^= 0.51)^a^			

Intercept	-7.30	30.3	n.s.

Ambulatory hospitalization rate per 1000 children in medstat	-0.02	0.27	n.s.

Length of stay	-0.71	1.3	n.s.

Ratio of boys to girls	64.8	25.0	**0.0115**

Ratio of Swiss to non Swiss residents	-0.58	0.86	n.s.

^a ^Coefficient of determination

## Discussion

This study shows that hospitalization rates in early childhood in Switzerland vary widely across gender, residential status, language regions and characteristics of residence. The highest hospitalization rates are found in urban or suburban areas and in the Italian part of Switzerland. The high ambulatory outpatient's hospitalization rate is significantly associated with lower stationary hospitalizations in the French part. More than 25% of all hospitalization discharges are due to respiratory problems, congruent with studies from U.S. [3,12].

Small-area analysis, a method to describe and analyze the utilization of health care in small geographic areas [medstat], allows the determination of variation in hospitalization rates between regions [7,8] and can be divided in supply- and demand-related factors. With the delineation of HSAP we propose the use of pediatric health service areas which are only partially related to cantonal borders [13,14], but represent, due to the complete data set of all hospitalizations in 4 years, effective user-based health markets for children, which is reflected in a fairly high localization index [9]. Hence, they form clustering units for the model-based analysis of potential determinants of health care utilization and provision concerning children's hospitalization. Its implementation into model based analysis allows an adequate interpretation of the effects of interest corrected for these clustering units.

### Demand related factors

Demand-related factors include language region, urbanization and population based factors. The higher hospitalization rate in the French and Italian speaking parts of Switzerland confirms well known cultural differences in Switzerland[15]. The perception of health as a consumable good might vary across Swiss language regions. The hurdle of bringing a child into a hospital is lower in the Italian and French parts of Switzerland, thus, with respective shorter length of stay for overall hospitalization. The model based analysis showed as well an association between the significant shorter lengths of stay in French parts of Switzerland and the respective higher hospitalization rates. However, in the Italian part, where highest hospitalization rates are present, no such significant association was found, when corrected for all other variables. Therefore, shorter LOS cannot compensate for the high hospitalization rates.

The difference of hospitalization rates between urban or suburban areas, village and rural areas refer to another demand-related approach. Beside the effect of facilitated access to hospital care in urban areas, environmental differences may play a substantial role in the aetiology or exacerbation of many conditions like asthma [12,16], bronchiolitis [17], gastroenteritis [18], or failure to thrive [19]. Numerous studies show an association of urban air pollution with higher hospitalization rates for respiratory diseases [20-22]. As shown in our data, higher hospitalization rates for respiratory problems are found in urban areas of all three language strata. However, more alarming is the fact that in the Italian part, famous for its moderate climate, not only the overall hospitalization rates are high but also those for respiratory problems are highest across all three urbanity types compared to the German and French parts. In addition, the LOS in Italian part, which is similar to the LOS of the French region, could indicate that the high hospitalization rates represent an effective need for problems of respiratory diseases assuming that LOS indicates in some degree the seriousness of disease. Environmental studies indicate that the highest concentrations of particles with a 50% cut-off aerodynamic diameter of 10 mm (PM10) and NO2 in the air are found in the Italian speaking part of Switzerland [23]. Therefore, analysis on stratified data with more information about air pollution in the different medstats has to be performed.

In contrast to a study about lower hospitalization rates of adult immigrants in Italy [24], the higher hospitalization rate of non-Swiss children can have manifold reasons. Non-medical factors like cultural differences, language problems, or even general uncertainty in a foreign country [25] as well as socio-economic differences between Swiss and non Swiss children can hold true for the observed higher hospitalization rates. Further studies on data analyzing subgroups of indications and patients are needed to confirm these hypotheses. Finally, the model based analysis could not confirm the unadjusted higher hospitalization rates for boys or a clear yearly trend towards higher hospitalization rates except for the German part.

### Supply related factors

The supply-related factors include availability and quality of health care related resources including hospitals and ambulatory care providers. It is more complex due to structural differences between different hospitals even within cantonal borders (e.g. degree of pediatric specialization, coding strategy [26], account system, availability of children's beds), which are only partially known. We could statistically account for such differences by including the HSAP in the model, which is one of the advantages using hospital service areas. Our study shows as well considerable differences in the range of provided ambulatory care. In the French speaking part, a higher supply of ambulatory outpatient clinics could slightly reduce the hospitalization rate, in the German and Italian parts no such association was found, possibly due to the fact that not enough outpatient facilities are provided by hospitals in general. Though, significant variation persists between the different HSAP. More studies have to analyze the association between utilisation on one side and density and quality of supply on the other side, in order to evaluate if the remaining variation is unwarranted or justified by underlying disease incidence.

### Strength and Limitations

The results of this study were derived from a complete coverage of stationary and ambulatory short stay hospitalizations of children (age <5 years of age) over 4 years and document for the first time the large heterogeneity in utilization and provision of young children's health care resources in Switzerland. The spatial model used in this study yielded hospital service areas in which, on average, 68.3% of the population used local health resources. We therefore consider this approach an appropriate spatial representation of utilisation and provision of hospitals in Switzerland. However, more structural information on hospital level (e.g. quality of care, availability of children's beds), on ambulatory care level (e.g. physician's density and speciality) and on medstat level (e.g. income as well as finer breakdown of political structure of medstat) is needed to draw relevant conclusions about the effect of supply and demand related factors on hospitalization rates. A further limitation derives from the fact that we could not disentangle hospitalizations from rehospitalisation, neither for stationary nor ambulatory hospitalization reasons. Further limitations concern the coding. For the stationary hospitalizations no data of the severity of disease was available. On the other hand the ambulatory hospitalizations vary substantially partially due to different coding systems in Switzerland [26].

## Conclusion

Out of a complete survey of hospitalization records over 4 years our study introduces the formation of an alternative spatial model of HSAP in Switzerland for relevant clustering effects of the small areas within population-based health markets and demonstrates a large heterogeneity in utilization and provision of young children's health care resources. We quantified the effects of urbanization and language regions as important elements in a complex health care system to account for variation in hospitalization rates for children. The results show that structural differences of HSAP account as well for some of the variation in hospitalization rates. However, further studies have to evaluate more in depth demand and supply-related factors for subgroups of indications in order to draw conclusions for potential adjustments of high hospitalization rates implying greater health care expenses.

## Competing interests

The authors declare that they have no competing interests.

## Authors' contributions

FSA wrote the manuscript and made all statistical analyses. MW and AB made contributions to the acquisition of data.

## Pre-publication history

The pre-publication history for this paper can be accessed here:


